# Efficacy of Rhythmic Photic Stimulation for Autonomic Nervous System Regulation in University Students

**DOI:** 10.33549/physiolres.935496

**Published:** 2025-02-01

**Authors:** Shang-Yu YANG, Pin-Chun WANG, Chin-Mao CHEN, Pin-Hsuan LIN, Cheng LIU

**Affiliations:** 1 Department of Healthcare Administration, College of Medical and Health Science, Asia University, Taichung, Taiwan; 2 Department of Nursing, Shu Zen Junior College of Medicine and Management, Kaohsiung, Taiwan; 3 Department of Occupational Therapy, College of Nursing and Health Sciences, Da-Yeh University, Changhua, Taiwan; 4 Department of Health and Beauty, Shu Zen Junior College of Medicine and Management, Kaohsiung, Taiwan; 5 Department of Physical Education, Huazhong University of Science and Technology, Wuhan, People’s Republic of China

**Keywords:** Binaural beat, Rhythmical photic stimulation, Autonomic nervous system, University student

## Abstract

University students frequently encounter stress and anxiety, impacting their autonomic nervous system and mental health. Rhythmic photic stimulation (RPS) at various frequencies is considered a potential intervention for anxiety and depression, but its effectiveness is not fully understood. This research aimed to assess the impact of RPS at theta (6 Hz), alpha (10 Hz), and beta (25 Hz) frequencies on autonomic nervous system regulation in university students, comparing the effects between those with and without depression symptoms. Conducted at a southern Taiwan university, this quasi-experimental study involved RPS interventions at specified frequencies, with pre and post assessments of heartbeat, blood pressure, and heart rate variability. Among 62 participants (average age 20.29±0.61), those without depression showed a notable blood pressure reduction following theta-frequency RPS compared to other frequencies (p<0.05). A similar pattern was observed when comparing non-depressed and depressed participants after theta-RPS, with depressed individuals experiencing an increase in sympathetic activity (p<0.05). RPS, particularly at theta frequency, can significantly influence the autonomic nervous system, suggesting a potential for reducing anxiety-related physiological markers in university students. Further verification with a larger and longitudinal study is warranted.

## Introduction

University students often encounter high stress and anxiety levels, adversely affecting their academic performance and quality of life. According to Shah and Pol [1], nearly half of university students experience symptoms of anxiety or depression. These mental health problems are attributable not only to academic pressure but also to the excessive use of social media [2,3], poor sleep quality [4–6], and concerns regarding career prospects [7]. Mental health problems are caused by the imbalance of the autonomic nervous system, especially overactivity of the sympathetic nervous system and reduction of parasympathetic nervous function, which are closely linked to the physiological mechanisms of anxiety and depression [8]. This imbalance leads to physiological responses such as accelerated heartbeat, increased blood pressure, and trouble sleeping, intensifying the psychological stress [9,10]. Therefore, exploring strategies to regulate the autonomic nervous system for improving the psychological health of university students is a critical concern in psychophysiology [11].

Several studies [12–14] have explored methods for managing and alleviating anxiety and depression, of which light therapy is one of the most promising. Humans are highly sensitive to light; even low-intensity light can alter melatonin levels to regulate circadian rhythms, heart rate variability (HRV), and the autonomic nervous system. For example, patients with seasonal affective disorder undergoing light therapy experience increased parasympathetic activity as feelings of relaxation [14]. A widely studied [12,13] form of light therapy is rhythmic photic stimulation (RPS), in which rhythmic visual stimuli are provided to the brain using strobe lights with adjustable frequency and intensity. This rhythmic stimulation leads to the synchronization of brain activity (brainwave frequency, neuronal synchronization, and desynchronization) to the rhythm of the stimuli, altering neural activity and physiological responses. RPS has been employed to alleviate symptoms of anxiety and depression, improve sleep, enhance cognition, and regulate emotions [12,13].

RPS is a technique involving flickering light at specific frequencies to entrain brain activity and promote specific physiological responses. Different frequencies of RPS – theta (6 Hz), alpha (10 Hz), and beta (25 Hz) – are known to correspond with various mental states such as relaxation, calmness, and focused alertness [12,13]. RPS operates by synchronizing brainwave frequencies with external light pulses, inducing a state of neural entrainment that modulates brainwave activity. This synchronization affects neural circuits within the prefrontal cortex and amygdala, key brain regions involved in emotional regulation and autonomic control [13,15]. By targeting these areas, RPS at specific frequencies may reduce stress responses and improve mood, contributing to the regulation of the autonomic nervous system and alleviating anxiety and depression symptoms[13, 15]. By presenting rhythmic flickers, RPS can synchronize neural oscillations with the stimulation frequency, potentially aiding in the regulation of the autonomic nervous system and addressing symptoms associated with anxiety and depression [12,13].

Kumano, Horie [16] used RPS interventions to treat depression and suggested that alpha-frequency (10 Hz) RPS can regulate HRV, improving mood. Additionally, Kim, Kim [13] observed the effects of alpha-frequency RPS intervention on mice with depression. Their study revealed that alpha-frequency RPS significantly improved anxiety, locomotor activity, social interaction, and despair behavior in the mice, with some of these improvements exceeding the therapeutic effects of fluoxetine (a medication for depression). Alpha waves are related to a relaxed brain state, and alpha-frequency RPS promotes favorable functional connectivity between brain areas responsible for emotional regulation and cognitive function, improving psychological and cognitive well-being. Moreover, low-frequency RPS can be used to treat seasonal affective disorder and nonseasonal depression [15]. Although RPS has been demonstrated to regulate HRV and reduce anxiety, more empirical research is required to verify its effectiveness and to determine the optimum therapeutic method.

University students experience numerous stressors, including academic stress, social pressure, career uncertainty, and emotional and psychological challenges. These stressors render university students particularly susceptible to anxiety symptoms [17–19], and they may require specialized interventions to support and maintain mental health [20,21]. Research has confirmed the benefits of RPS in regulating HRV, reducing depression, and decreasing anxiety [15,22]. However, the efficacy of RPS for regulating the autonomic nervous system is poorly understood. Furthermore, studies have primarily used the alpha frequency (10 Hz), and other frequencies (such as theta [6 Hz] and beta [25 Hz]) have remained unexplored.

The present study explored (1) the effectiveness of the RPS intervention at the theta, alpha, and beta frequencies for regulating the autonomic nervous system (reducing anxiety) of university students, (2) the differences in autonomic nervous system function among university students after the RPS intervention at these frequencies, and (3) the differences in autonomic nervous system function between university students with and without depression after the RPS intervention.

## Methods

### Study design and participants

This quasiexperimental trial was conducted at a central Taiwanese university from June to November 2023. Participants were enrolled into three intervention frequency groups: (A) theta, (B) alpha, and (C) beta. The order of interventions was randomized into six blocks (ABC, ACB, BAC, BCA, CAB, and CBA) to mitigate order effects, with a minimum 1-week washout period between interventions. The inclusion criteria were as follows: (1) being aged 20 years or older and (2) having no treatment for anxiety or depression during the intervention or in the 3 preceding months. The exclusion criteria were as follows: (1) having a psychiatric diagnosis by a physician, (2) experiencing a recent high-stress event, (3) having a history of epilepsy, and (4) having eye diseases. The study received ethical approval from the Research Ethics Committee of the China Medical University Hospital (CMUH110-REC3-021). The participants had given their written consent for their data to be used in this study.

### Intervention

The RPS intervention involved continuous flickering light delivered *via* specialized MindLightz glasses, which emitted light at three distinct frequencies: theta (6 Hz), alpha (10 Hz), and beta (25 Hz). Each participant received a 20-minute session of RPS at one of these specific frequencies per intervention, without alternating between frequencies during a session. The light was presented in a flicker format to induce brainwave entrainment, allowing brain activity to align with the chosen frequency. The light intensity was calibrated to a moderate level, and white light was utilized to maintain participant comfort and prevent overstimulation. This setup was intended to promote synchronization of brainwaves with the flickering light, potentially modulating the autonomic nervous system.

**Fig. 1:**
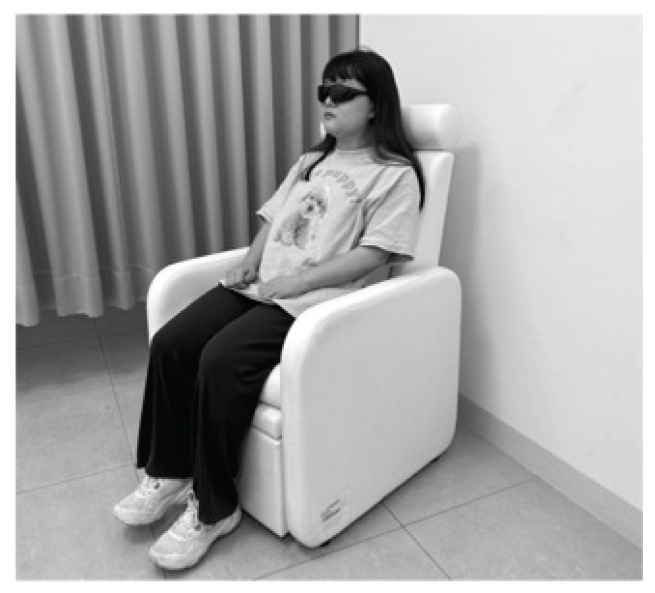
Participants received 20 min of RPS while seated and with eyes closed wearing RPS glasses.

Participants completed questionnaires for obtaining demographic information and the Chinese version of the Beck Depression Inventory (BDI), then rested for 10 min before preintervention measures of blood pressure and HRV were taken. The 20-minute intervention was followed by immediate postintervention measures. Interventions were performed between 9:00 AM and 12:00 PM in a controlled, quiet room, as illustrated in Figure 1. In each group, participants received 20 min of RPS while seated and with eyes closed wearing RPS glasses. MindLightz glasses and software were obtained from Mind Gear (Mentor, OH, USA). Additionally, at 8 min post intervention, researchers evaluated the participants for comfort, with any discomfort reported by participants leading to intervention termination.

### Measurements

Instrument measures of blood pressure and HRV were obtained to assess autonomic nervous system regulation. The demographic data collected comprised sex, age, religion, weekly exercise frequency, and perceived health status. The BDI measures depressive severity based on feelings over the previous 2 weeks on a Likert scale ranging from 0 to 3, with a total of 21 questions and a total score ranging from 0 to 63. Scores of 0–13, 14–19, 20–28, and 29–63 were categorized as normal, mild depression, moderate depression, and severe depression, respectively. Higher scores indicate higher depressive severity; the Chinese version of the BDI has strong psychometric properties. Blood pressure was measured using an Omron HEM-9700T (Omron, Kyoto, Japan). HRV was assessed using an HRV SA-3000p analyzer (Medicore, Seoul, Korea) to capture the standard deviation of NN intervals (SDNN), normalized low frequency (nLF), normalized high frequency (nHF), and low frequency/high frequency ratio (LF/HF). HRV was analyzed by calculating the low-frequency (LF) and high-frequency (HF) components. The LF component was defined within the frequency range of 0.04–0.15 Hz, while the HF component was set between 0.15–0.40 Hz, following standard HRV analysis guidelines [23]. An increase in SDNN indicates a strong autonomic regulation of the heart rate. nLF suggests increased sympathetic or reduced parasympathetic activity, whereas an increase in nHF indicates enhanced parasympathetic activity related to relaxation. The LF/HF ratio assesses the balance between the sympathetic and parasympathetic nervous systems.

### Data analysis

Data were analyzed using SPSS 22.0 for Mac (IBM, Armonk, NY, USA), with a significance level set at α=0.05. Descriptive statistics were calculated for participant demographics. Participants were categorized into nondepression (BDI: 0–13) and depression (BDI: 14+) groups for subgroup analyses. The Wilcoxon signed-rank test was used to compare within-group preintervention and postintervention differences in the heart rate, blood pressure (systolic/diastolic), and HRV parameters. The Friedman test was employed to examine between-group differences in score changes (posttest scores subtracted by pretest scores), and the Wilcoxon rank-sum test was employed to compare score changes between the groups postintervention.

## Results

### Participants

Demographic data of participants are presented in Table 1. This study included 5 men and 57 women with an average age of 20.29 years. The majority of participants had no religious affiliation, exercised 1–2 days per week, perceived themselves as having average health, and did not exhibit tendencies toward depression.

### Comparison of changes in heart rate, blood pressure, and HRV parameters before and after intervention

Median (interquartile range) values and Wilcoxon signed-rank test results for the heart rate, blood pressure, and HRV parameters are presented in Table 2. For participants without depression, significant reductions in systolic and diastolic blood pressure were observed in the theta group (p<0.05). The alpha group exhibited a significant decrease in systolic pressure (p<0.05) and an increase in SDNN and LF/HF (p<0.05). By The beta group exhibited a significant increase in SDNN (p<0.01). For participants with depression, significant increases in SDNN, nLF, and LF/HF values were observed in the theta group (p<0.01), as well as a significant decrease in nHF (p<0.01). The alpha group exhibited significant increases in the heart rate and nLF (p<0.05) and a decrease in nHF (p<0.01), and the beta group exhibited a significant decrease in diastolic pressure (p<0.05).

### Comparison of changes after each intervention

Friedman test results and median (interquartile range) score changes for the heart rate, blood pressure, and HRV parameters postintervention are presented in Table 3. For participants without depression, significant differences were found in systolic and diastolic blood pressure across the three groups (p<0.01). *Post hoc* analysis of the Wilcoxon signed-rank test results revealed that systolic pressure in the theta group was significantly lower than that in the beta group (p<0.05), and diastolic pressure in the theta group was significantly lower than that in the alpha and beta groups (p<0.05), suggesting that the theta group experienced a greater reduction in blood pressure.

### Comparison between participants with and without depression

The Wilcoxon rank-sum test results for comparisons of postintervention changes in the heart rate, blood pressure, and HRV parameters between participants with and without depression are presented in Table 4. Significant postintervention changes were observed in systolic pressure, diastolic pressure, nLF, and nHF in the theta group (p<0.05) and in diastolic pressure in the beta group (p<0.05). These results suggest that in the theta group, participants without depression exhibited a significantly greater reduction in blood pressure after the intervention compared with those with depression, who exhibited a significant increase in sympathetic nervous activity. In the beta group, a significantly greater increase in diastolic pressure was observed in participants with depression than in participants without depression.

## Discussion

This study employed RPS at varying frequencies to modulate the autonomic nervous system of university students. The results revealed that after a 20-minute session of RPS, most participants exhibited reduced blood pressure, improved autonomic system function, and increased sympathetic nerve activity. A comparison across the three frequencies showed that participants without depression exhibited a more significant reduction in blood pressure following theta-frequency RPS compared with alpha- or beta-frequency RPS. Additionally, participants without depression exhibited a greater decrease in blood pressure after theta-frequency RPS, whereas participants with depression experienced a more pronounced increase in sympathetic nerve activity.

The results presented in Table 2 demonstrate that participants without depression exhibited a decrease in blood pressure after theta-frequency and alpha-frequency RPS interventions, and after alpha-frequency and beta-frequency RPS, these participants also exhibited improved autonomic nervous function (as indicated by a significant increase in SDNN). The theta frequency may decrease regional cerebral blood flow, altering cerebral hemodynamics and affecting systemic blood pressure through the modulation of autonomic nervous system function [24]. Furthermore, because SDNN is an indicator of HRV and reflects overall changes in heart rate intervals, an increase in SDNN is a marker of strong autonomic system regulation and cardiovascular system function. RPS at alpha and beta frequencies may influence autonomic system regulation by stimulating specific brain regions, increasing SDNN [25]. The mechanisms underlying alpha-frequency and beta-frequency interventions in modulating the autonomic nervous system require further investigation.

The results presented in Table 2 reveal that participants with depression experienced an improvement in autonomic nervous system function and an increase in sympathetic nerve activity following the theta-frequency RPS intervention, and they also exhibited an increase in heart rate and sympathetic nerve activity following the alpha-frequency intervention, as well as a reduction in blood pressure following the beta-frequency intervention. Theta-frequency RPS may promote autonomic balance by regulating brain areas involved in emotional and stress responses, such as the prefrontal cortex and amygdala, contributing to enhanced sympathetic nerve activity (nLF) reflected in improvements in HRV metrics [26]. Alpha-frequency RPS may induce physiological changes in respiratory or cardiac rhythm adjustments. However, the changes in scores observed for participants with depression may be partially attributable to participants exhibiting anxiety or worry in response to the interventions, triggering physiological responses such as accelerated heart rate. Finally, the beta frequency is typically associated with alertness and increased attention and may elicit a relaxation response, leading to a decrease in blood pressure in participants with depression [27].

The interpretation of nLF as an indicator of autonomic balance is complex due to its sensitivity to both sympathetic and parasympathetic activity. In this study, we noted changes in nLF values following RPS at various frequencies, and while nLF is often interpreted to indicate increased sympathetic or decreased parasympathetic modulation, this interpretation remains subject to limitations. Specifically, nLF in normalized units represents the proportion of LF power relative to the combined power of both LF and HF components, after subtracting VLF (very low frequency) power from the total spectral power [23]. Therefore, rather than indicating absolute sympathetic activity, nLF should be interpreted as reflecting a shift in autonomic balance, which may lean towards sympathetic or parasympathetic modulation depending on other contextual markers (such as heart rate variability and SDNN). Further studies using additional physiological indicators would be needed to delineate these contributions with greater precision.

The results in Table 3 indicate that participants without depression had a more significant decrease in blood pressure following theta-frequency RPS compared with RPS at the other frequencies, a phenomenon not observed in participants with depression. The theta frequency is commonly associated with relaxation and increased calmness, ensuring the balance of the autonomic nervous system and decreasing blood pressure [28]. Additionally, physiological differences may exist between participants with and without depression that affect their response to the intervention. Theta-frequency RPS may be more effective in modulating the autonomic nervous system and reducing the blood pressure of participants without depression [29]. Depression itself may affect autonomic nervous system function, resulting in a distinct physiological response to theta-frequency RPS in individuals with depression.

The results of the present study (Table 4) demonstrated that following theta-frequency RPS, participants without depression exhibited a significantly greater reduction in blood pressure compared with participants with depression; however, participants with depression exhibited a more substantial increase in sympathetic nerve activity. These results suggest that theta-frequency RPS produces more pronounced effects in individuals without depression, effectively inducing relaxation and reducing sympathetic nervous system activity, leading to significant reductions in blood pressure. By contrast, individuals with depression may experience altered or attenuated physiological responses to theta-frequency RPS interventions due to the chronic stress and dysregulation of the autonomic nervous system associated with depressive disorders. Furthermore, theta-frequency RPS promotes relaxation and calm by regulating brainwave activity. For individuals without depression, this stimulation may enhance parasympathetic activity, reduce sympathetic activity, and cause relaxation and decreased blood pressure. However, for those with depression, compromised autonomic regulatory mechanisms induce a distinct response to theta-frequency RPS, perhaps because their nervous systems are more sensitive or have higher levels of sympathetic activity [30], resulting in a significant increase in sympathetic nerve activity post-RPS.

Following the administration of RPS at the beta frequency, participants with depression exhibited a significant increase in blood pressure compared with participants without depression (Table 4). Beta waves are brainwave frequencies associated with alertness, attention, focused thinking, and emotional regulation. Under normal conditions, an increase in beta wave activity is associated with enhanced cognitive activity and emotional processing [31]. The nervous system of individuals with depression may be more sensitive to stimulation at the beta frequency, which may induce a state of heightened alertness and stress [32]. This increased state of alertness may increase blood pressure by enhancing the activity of the sympathetic nervous system. However, further research is required to explore the mechanisms and long-term effects of beta-frequency RPS in individuals with depression.

The results summarized in Tables 2, 3, and 4 indicate that while both theta and alpha frequencies impacted blood pressure and autonomic activity, theta frequency demonstrated a more consistent effect in reducing blood pressure among non-depressed participants and modifying autonomic responses. Specifically, although alpha frequency also led to a significant decrease in blood pressure and increased sympathetic activity in depressed participants, the effects of theta frequency were more pronounced and consistent across various HRV parameters. This suggests that theta frequency may have a distinctive role in autonomic modulation, though the overlapping effects observed with alpha frequency warrant further investigation.

This study has several limitations. First, participants were all from a single university in southern Taiwan, and the sample size was small, limiting the interpretive strength of the results. Second, other demographic variables, such as dietary habits, may influence the heart rate, blood pressure, and HRV parameters. Third, the intervention duration was set to 20 min, which may have been insufficient to produce results, and the effects of RPS were assessed without long-term follow-up data for determining persistent effects. Future research should extend the intervention duration and include continual tracking measurements. Future studies with an increased sample size and a randomized controlled trial design are required to reduce the occurrence of bias. Fourth, the interpretation of the LF/HF ratio as a marker of sympathovagal balance remains debated. Initially, this ratio was based on 24-hour HRV recordings to estimate the balance between sympathetic (LF) and parasympathetic (HF) activity [33]. However, research suggests that LF may represent both sympathetic and parasympathetic influences, which complicates using LF/HF as a definitive indicator, particularly in short-term recordings like those used in this study [34]. Additional markers could provide more precise insights in future research. Fifth, this study used a one-week wash-out period between RPS interventions, based on studies showing that photic stimulation effects generally dissipate within hours to days [35,36]. While this period likely minimized carry-over effects, we acknowledge that the optimal wash-out duration may vary. Future research could consider a longer wash-out period for added robustness. Despite these limitations, this study demonstrated the effectiveness of RPS interventions at various frequencies for regulating the autonomic nervous system in university students, comparing the results of the intervention in participants with and without depression. The results can provide a reference for the future use of RPS interventions to address anxiety and depression in university students.

## Conclusions

The results of this study indicate that RPS at specific frequencies exert significant effects on the regulation of the autonomic nervous system in university students. Specifically, the theta frequency significantly reduced blood pressure in participants without depression, and a significant increase in sympathetic nervous activity was observed in participants with depression after the theta-frequency intervention. Although alpha frequency showed similar effects, theta frequency’s impact was more consistent. These findings highlight the potential of RPS as a nonpharmacological intervention for regulating the autonomic nervous system and improving physiological indicators related to anxiety and depression. However, these results are preliminary and further studies with larger and more diverse samples are essential to validate these findings and clarify the unique contributions of each frequency.

## Figures and Tables

**Table 1:** Participant demographic characteristics (*N*=62).

Demographic characteristics	n (%)
*Sex*	
* Male*	5 (8.1)
* Female*	57 (91.9)
*Age, mean± SD (year-old)*	20.29 ± 0.61
*Religion*	
* No*	41 (66.1)
* Yes*	21 (33.9)
*Exercise per week (day)*	
* 0*	13 (21.0)
* 1–2*	27 (43.5)
* ≧3*	22 (35.4)
*State of perceived health*	
* Poor*	5 (8.1)
* Fair*	35 (56.5)
* Good*	22 (35.5)
*BDI, mean ± SD (score)*	10.31 ± 8.99
* Normal (0–13)*	44 (71.0)
* Mild depression (14–19)*	12 (19.4)
* Moderate depression (20–28)*	2 (3.2)
* Severe depression (29–63)*	4 (6.5)

SD: standard deviation. BDI: Beck Depression Inventory.

**Table 2:** Heart rate, blood pressure, and heart rate variability (HRV) parameters before and after each intervention, stratified by depression status.

	Theta (6 Hz)			Alpha (10 Hz)			Beta (25 Hz)		
*Outcome variable*	Pre-test	Post-test	p-value	Pre-test	Post-test	p-value	Pre-test	Post-test	p-value
	Median (IQR)	Median (IQR)		Median (IQR)	Median (IQR)		Median (IQR)	Median (IQR)	
*Non-depressed participants (n=44)*									
*HR (beats/min)*	77.50 (73.00, 85.50)	79.00 (69.00, 84.75)	0.06	78.50 (71.00, 84.00)	80.00 (71.50, 86.00)	0.70	78.00 (69.50, 86.00)	80.00 (72.00, 86.00)	0.60
*Systolic blood pressure (mm Hg)*	106.00 (101.00, 109.75)	100.50 (93.50, 111.50)	<0.01*	104.00 (97.25, 112.00)	102.00 (96.50, 108.00)	0.02*	104.00 (98.00, 109.50)	104.00 (96.00, 112.00)	0.55
*Diastolic blood pressure (mm Hg)*	65.50 (61.00, 70.75)	62.00 (57.25, 70.00)	0.02*	61.50 (57.25, 70.50)	61.00 (58.00, 68.00)	0.91	63.50 (58.00, 71.00)	64.00 (59.00, 71.00)	0.49
*SDNN*	53.03 (41.07, 65.15)	54.12 (40.48, 68.81)	0.15	46.67 (33.49, 63.32)	50.38 (41.03, 66.81)	<0.01*	46.67 (36.61, 56.86)	53.43 (40.57, 63.95)	<0.01*
*nLF*	67.61 (54.10, 75.98)	65.96 (51.22, 81.45)	0.31	60.79 (46.85, 72.03)	67.75 (46.48, 75.97)	0.12	62.55 (46.36, 75.59)	64.56 (51.00, 81.30)	0.27
*nHF*	32.28 (24.41, 50.62)	34.04 (18.55, 48.78)	0.23	38.74 (26.37, 53.09)	32.74 (24.03, 53.52)	0.15	37.45 (24.41, 53.64)	35.44 (18.70, 49.00)	0.27
*LF/HF*	2.01 (0.98, 3.10)	1.94 (1.05, 4.39)	0.35	1.55 (0.88, 2.58)	2.10 (0.92, 3.16)	0.05*	1.67 (0.86, 3.10)	1.83 (1.04, 4.35)	0.32
*Depressed participants (n=18)*									
*HR (beats/min)*	77.00 (71.25, 85.50)	75.00 (68.50, 80.50)	0.35	77.00 (64.75, 80.00)	81.00 (63.75, 85.00)	0.05*	75.00 (69.25, 86.25)	75.00 (71.00, 82.00)	0.41
*Systolic blood pressure (mm Hg)*	102.00 (99.00, 105.00)	102.00 (98.50, 107.75)	0.67	101.00 (99.50, 106.50)	101.00 (99.75, 103.75)	0.60	102.00 (94.75, 108.75)	102.00 (96.00, 105.00)	0.30
*Diastolic blood pressure (mm Hg)*	62.00 (58.75, 67.50)	64.00 (61.00, 67.00)	0.43	59.00 (57.00, 67.50)	64.00 (57.50, 69.25)	0.87	59.00 (53.00, 60.50)	58.00 (56.00, 67.25)	0.02*
*SDNN*	52.90 (35.72, 55.86)	56.80 (45.86, 69.34)	<0.01*	58.67 (39.29, 66.25)	59.81 (40.72, 81.45)	0.11	50.30 (35.57, 66.85)	54.30 (34.58, 78.12)	0.47
*nLF*	48.99 (39.63, 62.94)	63.93 (56.28, 76.81)	<0.01*	59.17 (36.09, 74.71)	68.09 (53.20, 82.50)	0.02*	51.67 (38.42, 71.22)	56.20 (47.07, 72.06)	0.11
*nHF*	51.01 (37.06, 60.37)	36.07 (23.19, 43.58)	<0.01*	40.83 (25.29, 63.92)	31.91 (17.50, 46.80)	0.02*	48.33 (28.78, 61.58)	43.80 (27.94, 52.93)	0.11
*LF/HF*	0.96 (0.66, 1.70)	1.77 (1.29, 3.32)	<0.01*	1.45 (0.57, 3.22)	2.13 (1.16, 4.78)	0.09	1.07 (0.63, 2.47)	1.28 (0.89, 2.60)	0.16

SD: standard deviation. HR: heart rate. SDNN: standard deviation of all R-R [NN] intervals. nLF: normalized low frequency. nHF: normalized high frequency. p-value: Wilcoxon signed-rank test;

* p<0.05.

**Table 3:** Changes in heart rate, blood pressure, and HRV parameters after each intervention, stratified by depression status.

	Theta (6 Hz)	Alpha (10 Hz)	Beta (25 Hz)		
*Outcome variable*	Median (IQR)	Median (IQR)	Median (IQR)	p-value	*Post hoc*
*Non-depressed participants (n=44)*					
*HR (beats/min)*	−2.00 (−7.00, 3.00)	0.00 (−4.75, 6.00)	−1.00 (−5.75, 4.75)	0.08	
*Systolic blood pressure (mm Hg)*	−6.00 (−10.75, −0.25)	−2.00 (−5.75, 2.75)	−1.50 (−5.00, 3.75)	<0.01*	6 Hz<25 Hz
*Diastolic blood pressure (mm Hg)*	−2.00 (−8.00, 1.75)	0.00 (−4.00, 3.00)	0.50 (−3.00, 4.00)	<0.01*	6 Hz<10 Hz; 6 Hz<25 Hz
*SDNN*	1.95 (−4.35, 12.55)	4.38 (−2.01, 14.50)	7.19 (−1.92, 14.06)	0.23	
*nLF*	1.31 (−7.43, 16.60)	4.66 (−10.83, 17.82)	4.89 (−9.66, 13.77)	0.85	
*nHF*	−1.31 (−16.60, 6.43)	−3.44 (−17.54, 10.83)	−4.90 (−13.77, 9.66)	0.91	
*LF/HF*	0.40 (−0.95, 1.23)	0.27 (−0.43, 1.45)	0.34 (−0.72, 1.37)	0.78	
*Depressed participants (n=18)*					
*HR (beats/min)*	−2.00 (−5.75, 3.25)	2.00 (0.00, 5.25)	0.00 (−2.75, 7.25)	0.12	
*Systolic blood pressure (mm Hg)*	0.00 (−2.00, 3.25)	0.00 (−3.25, 1.50)	1.00 (−2.00, 8.50)	0.98	
*Diastolic blood pressure (mm Hg)*	2.00 (−2.00, 3.00)	0.00 (−3.25, 1.50)	5.00 (0.75, 9.25)	0.21	
*SDNN*	7.03 (−2.47, 16.45)	12.37 (−2.31, 16.92)	4.00 (−4.81, 8.89)	0.90	
*nLF*	17.43 (4.88, 24.67)	8.77 (2.90, 19.78)	5.80 (−5.26, 18.76)	0.80	
*nHF*	−17.43 (−24.72, −4.88)	−8.77 (−19.78, −2.91)	−5.80 (−18.76, 5.26)	0.80	
*LF/HF*	0.93 (0.22, 1.70)	1.02 (0.01, 2.67)	0.35 (−0.36, 1.13)	0.41	

SD: standard deviation. HR: heart rate. SDNN: standard deviation of all R-R [NN] intervals. nLF: normalized low frequency; nHF: normalized high frequency. p-value: Friedman test. *Post hoc*: Wilcoxon signed–rank test.

**Table 4:** Changes in heart rate, blood pressure, and HRV parameters after each intervention for participants with and without depression.

	Theta (6 Hz)			Alpha (10 Hz)			Beta (25 Hz)		
	Non-depression	Depression		Non-depression	Depression		Non-depression	Depression	
*Outcome variable*	Median (IQR)	Median (IQR)	p-value	Median (IQR)	Median (IQR)	p-value	Median (IQR)	Median (IQR)	p-value
*HR (beats/min)*	−2.00 (−7.00, 3.00)	−2.00 (−5.75, 3.25)	0.72	0.00 (−4.75, 6.00)	2.00 (0.00, 5.25)	0.28	−1.00 (−5.75, 4.75)	0.00 (−2.75, 7.25)	0.29
*Systolic blood pressure (mm Hg)*	−6.00 (−10.75, −0.25)	−0.00 (−2.00, 3.25)	<0.01*	−2.00 (−5.75, 2.75)	0.00 (−3.25, 1.50)	0.37	−1.50 (−5.00, 3.75)	1.00 (−2.00, 8.50)	0.17
*Diastolic blood pressure (mm Hg)*	−2.00 (−8.00, 1.75)	2.00 (−2.00, 3.00)	0.04*	0.00 (−4.00, 3.00)	0.00 (−3.25, 1.50)	0.88	0.50 (−3.00, 4.00)	5.00 (0.75, 9.25)	0.03*
*SDNN*	1.95 (−4.35, 12.55)	7.03 (−2.47, 16.45)	0.42	4.38 (−2.01, 14.50)	12.37 (−2.31, 16.92)	0.80	7.19 (−1.92, 14.06)	4.00 (−4.81, 8.89)	0.29
*nLF*	1.31 (−7.43, 16.60)	17.43 (4.88, 24.67)	0.04*	4.66 (−10.83, 17.82)	8.77 (2.90, 19.78)	0.32	4.89 (−9.66, 13.77)	5.80 (−5.26, 18.76)	0.37
*nHF*	−1.31 (−16.60, 6.43)	−17.43 (−24.72, −4.88)	0.04*	−3.44 (−17.54, 10.83)	−8.77 (−19.78, −2.91)	0.26	−4.90 (−13.77, 9.66)	−5.80 (−18.76, 5.26)	0.37
*LF/HF*	0.40 (−0.95, 1.23)	0.93 (0.22, 1.70)	0.08	0.27 (−0.43, 1.45)	1.02 (0.01, 2.67)	0.35	0.34 (−0.72, 1.37)	0.35 (−0.36, 1.13)	0.64

IQR: interquartile range. HR: Mean heart rate. SDNN: standard deviation of all R-R intervals. nLF: normalized low frequency; nHF: normalized high frequency. p-value: Wilcoxon rank-sum test;

* p<0.05.
